# Mast Cells Positive to Tryptase and c-Kit Receptor Expressing Cells Correlates with Angiogenesis in Gastric Cancer Patients Surgically Treated

**DOI:** 10.1155/2013/703163

**Published:** 2013-11-20

**Authors:** Michele Ammendola, Rosario Sacco, Giuseppe Sammarco, Giuseppe Donato, Valeria Zuccalà, Roberto Romano, Maria Luposella, Rosa Patruno, Carlo Vallicelli, Giorgio Maria Verdecchia, Davide Cavaliere, Severino Montemurro, Girolamo Ranieri

**Affiliations:** ^1^Department of Medical and Surgery Science, Clinical Surgery Unit, University of Catanzaro “Magna Graecia” Medical School, Viale Europa, Germaneto, 88100 Catanzaro, Italy; ^2^Health Science Department, Pathology Unit, University of Catanzaro “Magna Graecia” Medical School, 88100 Catanzaro, Italy; ^3^Department of Medical and Surgery Science, Cardiovascular Disease Unit, University of Catanzaro “Magna Graecia” Medical School, 88100 Catanzaro, Italy; ^4^Surgery Unit, National Cancer Research Centre, Giovanni Paolo II, 70100 Bari, Italy; ^5^Department of Surgery, Surgical Oncology Unit, Ospedale Morgagni-Pierantoni, 47121 Forlì, Italy; ^6^Interventional Radiology Unit with Integrated Section of Translational Medical Oncology, National Cancer Research Centre, “Giovanni Paolo II”, 70100 Bari, Italy

## Abstract

*Background*. Angiogenesis is a complex process involved in both growth and progression of several human and animal tumours. Tryptase is a serin protease stored in mast cells granules, which plays a role in tumour angiogenesis. Mast cells (MCs) can release tryptase following c-Kit receptor (c-KitR) activation. *Method*. In a series of 25 gastric cancer patients with stage T_3_N_2-3_M_0_ (by AJCC for Gastric Cancer 7th Edition), immunohistochemistry and image analysis methods were employed to evaluate in the tumour tissue the correlation between the number of mast cells positive to tryptase (MCPT), c-KitR expressing cells (c-KitR-EC), and microvascular density (MVD). *Results*. Data demonstrated a positive correlation between MCPT, c-KitR-EC, and MVD to each other. In tumour tissue the mean number of MCPT was 15, the mean number of c-KitR-EC was 20, and the mean number of MVD was 20. The Pearson test correlating MCPT and MVD, c-KitR-EC and MVD was significantly (*r* = 0.64, *P* = 0.001; *r* = 0.66, *P* = 0.041, resp.). *Conclusion*. In this pilot study, we suggest that MCPT and c-KitR-EC play a role in gastric cancer angiogenesis, so we think that several c-KitR or tryptase inhibitors such as gabexate mesilate and nafamostat mesilate might be evaluated in clinical trials as a new antiangiogenetic approach.

## 1. Introduction

Angiogenesis is a complex process involved in growth, invasion, and metastasis of several animal and human tumours [1–3]. Mast cells (MCs) intervene in this process releasing classical proangiogenic factors, such as vascular endothelial growth factor (VEGF), thymidine phosphorylase (TP), fibroblast growth factor-2 (FGF-2), and nonclassical proangiogenic factors, such as tryptase and chymase, stored in their secretory granules [4–8]. The role of MCs has been broadly studied in benign lesions, in animal's and human's cancers, such as keloids, mast cells tumours, head and neck, colorectal, lung, and cutaneous malignancies, indicating that MCs density is highly correlated with the extent of tumour angiogenesis [9–13]. Recent data have shown that MCs density is correlated with angiogenesis and progression of patients with gastric carcinoma [14, 15]. However, no data have been published regarding the correlation between MCs positive to tryptase (MCPT), c-Kit receptor expressing cells (c-KitR-EC), and microvascular density (MVD) in gastric carcinoma tissue. In the present study, we have evaluated correlations between the number of MCPT, c-KitR-EC, and MVD in a series of 25 gastric carcinomas with stage T_3_N_2-3_M_0_ (by AJCC for Gastric Cancer 7th Edition), by means of immunohistochemistry and image analysis methods.

## 2. Methods

### 2.1. Patients

The clinicopathological features of studied patients are summarized in Table 1. A total of 25 gastric cancer patients diagnosed with preoperative gastric endoscopy underwent curative resection. Surgical approach used was open total gastrectomy with D2 lymph node dissection [16]. Patients were staged according to the American Joint Committee on Cancer 7th edition (AJCC-TNM) classification [17]. We have selected patients with stages III A and III B to the aim to correlate if MCPT, c-KitR-EC, and MVD in primary tumour tissue were associated with nodal involvement. On the other hand, metastatic patients with stage IV have no indication of surgery [13]. All patients had no distant metastases on computed tomography (TC) of the thorax, abdomen, and pelvis. All samples evaluated in this study were of adenocarcinomas histological type. Full ethical approval and consent from individual patients were obtained to conduct the study.

### 2.2. Immunohistochemistry

For the evaluation of MCPT, c-KitR-EC, and MVD, a three-layer biotin-avidin-peroxidase system was utilized [18]. Briefly, 4 *μ*m thick serial sections of formalin-fixed and paraffin-embedded tumour samples were deparaffinised. Then, for antigen retrieval, sections were microwaved at 500 W for 10 min, after which endogenous peroxidase activity was blocked with 3% hydrogen peroxide solution. Next, adjacent slides were subsequently incubated with monoclonal antibodies anti-CD31 (clone JC70a; Dako) diluted 1 : 40 for 30 min and pH 8 at room temperature, anti-c-KitR (CD117; Dako) for 30 min and pH 8, and anti-tryptase (clone 10D11; Novo Castra) diluted 1 : 150 for 20 min and pH 6 at room temperature. The bound antibody was visualized using biotinylated secondary antibody, avidin-biotin peroxidase complex, and 3-amino-9-ethylcarbazole. Nuclear counterstaining was performed with Gill's haematoxylin no. 2 (Polysciences, Warrington, PA, USA).

### 2.3. Morphometrical Assay

An image analysis system (Semiquantimet 400 Nikon) has been used. MCPT, c-KitR-EC, and MVD were observed at low magnification, and “hot spots” were selected at ×200 magnification [1, 17]. Areas of necrosis were not considered for counting. Hot spots were evaluated in three serial sections, and each single MCPT, c-KitR-EC, MVD was counted at ×400 magnification and reported as media from sections in order to avoid possible variability between sections.

### 2.4. Statistical Analysis

Linear correlations between groups were quantified by means of the Pearson's correlation coefficient (*r*). *t*-test was used to statistically compare means. Correlation among MCPT, c-KitR-EC, MVD, lymph nodal involvement and the main clinical pathologial features were analysed by chi-square test. *P* < 0.05 was considered significant. All statistical analyses were performed with the SPSS statistical software package (SPSS, Inc., Chicago, IL).

## 3. Results

**Figure 1:**
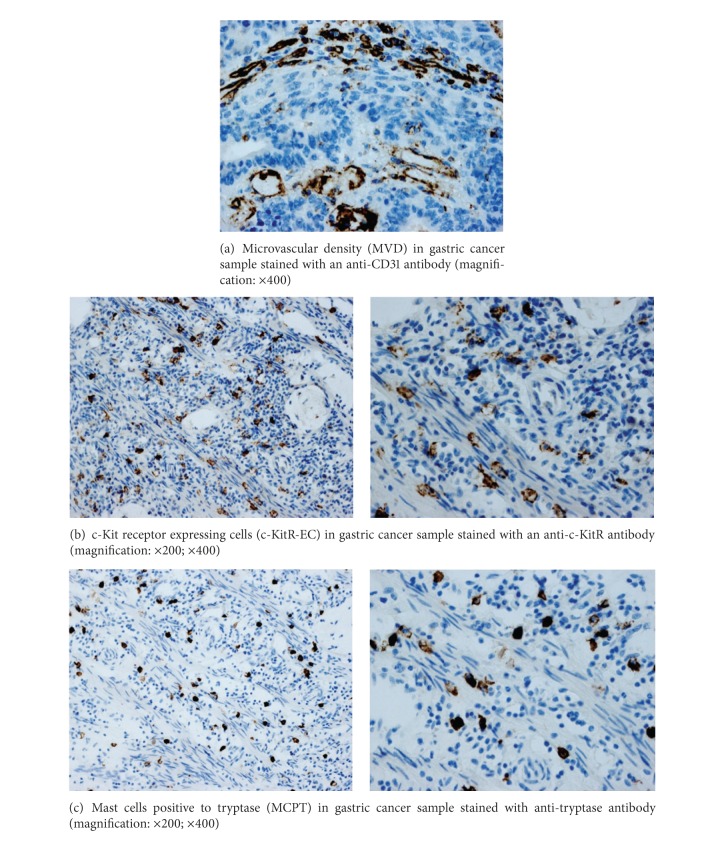
Representative immunohistochemical images relative to MCPT, c-KitR-EC, and MVD in gastric cancer tissue. Scale bar represents 100 *μ*m.

**Figure 2:**
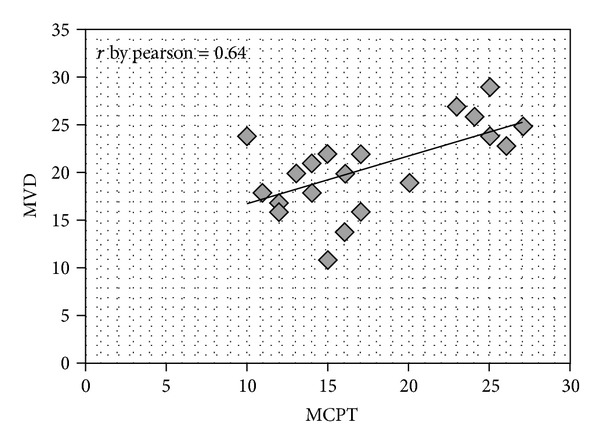
Pearson distribution of correlation between MCPT and MVD *r* = 0.64.

**Figure 3:**
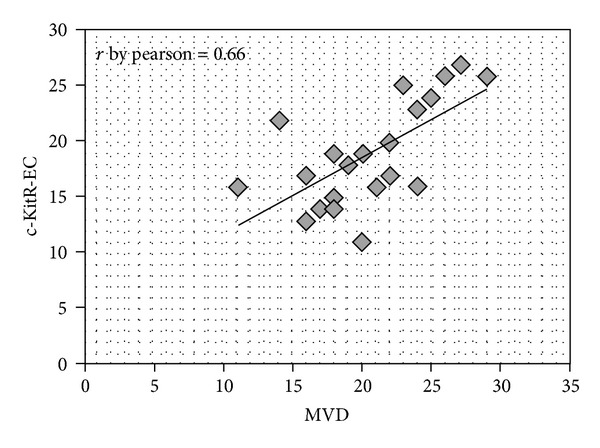
Pearson distribution of correlation between c-KitR-EC and MVD *r* = 0.66.

Immunohistochemical staining by antibodies anti-tryptase, anti-c-KitR and anti-CD31 allows the demonstration that MCPT and c-KitR-EC are well recognizable in highly vascularized gastric carcinoma tissue (Figure 1). Due to the possible interobserver variability at light microscopy in the evaluation of MCPT, c-KitR-EC, and MVD, the counts were performed by mean the above image analysis system. It is important to underline that the hot spots were evaluated at ×400 magnification in a well-reproducible microscopic area of 0.019 mm^2^. In this manner, our results are related to an identified microscopic area. Considering all 25 samples (Table 1), in tumour tissue, the mean number of MCPT was 15, the mean number of c-KitR-EC was 20, and the mean number of MVD was 20. The Pearson test correlating MCPT and MVD and c-KitR-EC and MVD was significantly (*r* = 0.64, *P* = 0.001; *r* = 0.66, *P* = 0.041, resp.) (Figures 2 and 3, resp.). In the present study, clinicopathological features of patients were analysed, but no correlation among MCPT, c-KitR-EC, MVD, lymph nodal involvement and the main clinicopathological features was found.

## 4. Discussion

Up to now, the role of MCs in gastric cancer angiogenesis has not been clarified completely. We have a lot of data about the angiogenic process and its drug targets in tumours [19, 20], but there are few data on the role of MCs in gastric cancer angiogenesis [14, 21]. In particular, in a study designed by Mukherjee et al. [22], the authors studied MCs density in patients with gastric ulcers, well-differentiated cancers, and poorly differentiated cancers. The study was performed on biopsies from gastric ulcers, well-differentiated cancers, and poorly differentiated cancers by means of toluidine blue stain. In this study, MCs density in well-differentiated was much higher than poorly differentiated carcinoma and correlated with angiogenesis.

Ribatti et al. [14] studied tumour samples from gastric cancer patients by means of immunohistochemistry employing anti-tryptase and anti-chymase antibodies to stain MCs found. In this study, a correlation between microvascular density and tryptase and chymase-positive masts cells with histopathological type was found.

Differences between the above studies and our results may be explained on the basis of different methods to identify MCs (toluidine blue, anti-tryptase antibody, and anti-chymase antibody), methods to assess MCs count (hot spots, random fields, and magnification), type of studied tissue (biopsy or surgically resected tumour), and stage of disease.

Taken together, these studies suggest that MCs are involved in gastric cancer angiogenesis. It is well demonstrated that tryptase is one of the most powerful angiogenic mediators released by human MCs following c-KitR activation and it may be angiogenic via several mechanisms. Tryptase is involved in tissue remodelling, and it may also act indirectly on tissue neovascularization by releasing latent angiogenic factors bound to the extracellular matrix [23–26]. Tryptase is an agonist of the proteinase-activated receptor-2 (PAR-2) in vascular endothelial cells. Activation of PAR-2 induces cell proliferation and the release of IL-6 and granulocyte-macrophage colony stimulating factor (GM-CSF), which, in turn, acts as angiogenic molecule [27]. 

In this pilot study, we found in a series of 25 surgical gastric cancer patients that MCPT and c-KitR-EC in tumour tissue, regardless of tumour staging or site, are positively correlated to the MVD. Our preliminary data suggest that MCPT and c-KitR-EC might play a role in gastric cancer angiogenesis. Further study in a large series of patients will be necessary to confirm first results. It is of interest underline that in this context, several c-KitR inhibitors such as imatinib mesilate or tryptase inhibitors such as gabexate mesilate and nafamostat mesilate [28, 29] might be evaluated in clinical trials as new antiangiogenetic strategy.

## Figures and Tables

**Table 1:** Clinicopathological features of patients.

	*N*
Overall series	25
Age	
<65	8
>65	17
Sex	
Male	15
Female	10
Tumour site	
Cardia	5
Lesser curvature	3
Greater curvature	4
Body and fundus	7
Pyloric area	6
TNM by AJCC stage and type by Lauren classification	
T_3_N_2_M_0_	14
T_3_N_3_M_0_	11
Intestinal type	16
Diffuse type	9
Histologic type	
Adenocarcinomas	25
Histologic grade	
G1	3
G2	8
G3	14
